# Supplementary material for: Food Risk Analysis: Towards a Better Understanding of “Hazard” and “Risk” in EU Food Legislation
Source: Foods. 2023 Jul 27;12(15):2857. doi: 10.3390/foods12152857 (PMC10418315; doi:10.3390/foods12152857)
Supplement: Supplementary file 1 [file foods-12-02857-s001.zip › Supplementary Material 3/CS/meet-our-current-fellows-2021-2022.pdf]

5th European Food Risk Assessment Fellowship cohort 2021-2022

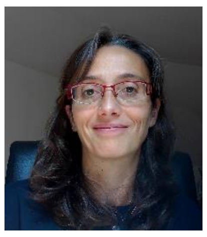

Cristina Alonso Andicoberry, EU-FORA Programme Manager

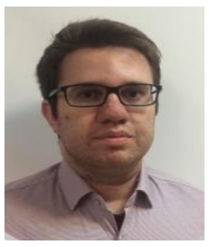

Plamen Panayotov, EFSA Trainee

Contact: EU-FORA@efsa.europa.eu

HOSTING ORGANISATION	COUNTRY	WORK PROGRAMME	FELLOW	FROM
Risiken erkennen – Gesundheit schützen<br>**Federal Institute for Risk Assessment (BfR)**	Germany	The use of NAM and omics data in risk assessment	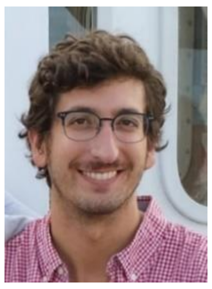<br>**Andrea Miccoli**	Italy
		Insects in food and their relevance regarding allergenicity assessment	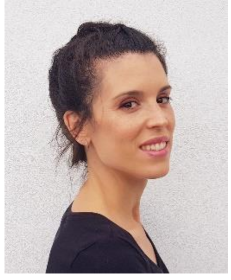<br>**Lidia Delgado Calvo-Flores**	Spain
		Risk Assessment of Food Contact Materials	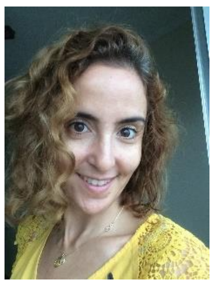<br>**Otilia Carvalho**	Portugal
INSTITUTE OF AGRICULTURAL AND FOOD BIOTECHNOLOGY<br>**Institute of Agriculture and food biotechnology (IBPRS-PIB)**	Poland	Risk assessment of contaminants in foods retailed by a large international food distributor in Poland.	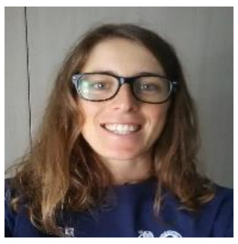<br>**Chiara Balbo**	Italy
		Microbiological risk assessment of traditional food of animal origin produced in short supply chains in Poland.	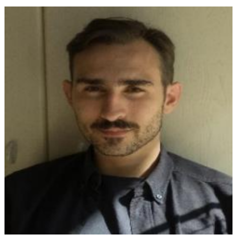<br>**Constantine Richard Stefanou**	Greece

HOSTING ORGANISATION	COUNTRY	WORK PROGRAMME	FELLOW	FROM
**Institute of Marine Research (IMR) & Norwegian Institute of Public Health (NIPH)**	Norway	Developing a framework for open and FAIR data management practices for next generation risk- and benefit assessment of fish and seafood	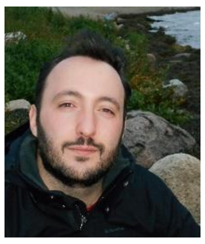 **Javier Pineda Pampliega**	Spain
**Instituto Nacional de Investigaciòn y Tecnologìa agraria y alimentaria (INIA)**	Spain	Impact of drinking water treatment processes on the residues of plant protection products for consumer risk assessment. Theoretical and experimental studies.	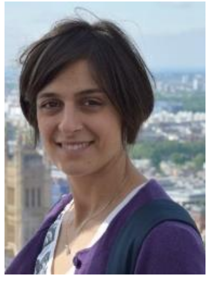 **Angela Mari**	Italy
**National Food Institute, Technical University of Denmark (DTU Food)**	Denmark	Allergenicity risk assessment	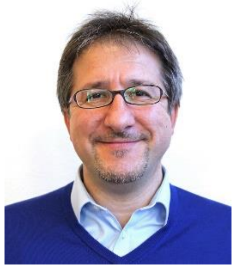 **Biase Liguori**	Italy
**National Research Council of Italy Institute of biochemistry and cell biology (CNR-IBBC)**	Italy	Environmental Modifiers causing Neurodegeneration (EMOgen)	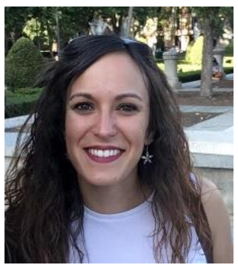 **Ana Guillem Amat**	Spain
		Risk assessment of honeybee stressors based on in silico analysis of molecular interactions	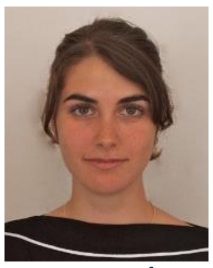 **Monica del Águila**	Spain
		Use of biosensors for rapid and sensitive detection of pesticides in food samples for Food Safety Chemical Risk Assessment.	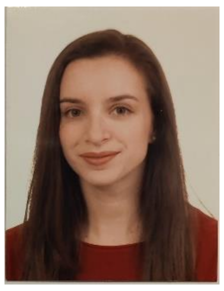 **Vasiliki Garefalaki**	Greece
**National Research Council of Italy Institute of Sciences of Food Productions (CNR-ISPA)**	Italy	Risk Assessment/Risk Communication: understanding the context and addressing Priorities of the future — a learning-by-doing approach	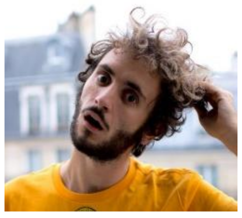 **Frederic Bayer**	France

HOSTING ORGANISATION	COUNTRY	WORK PROGRAMME	FELLOW	FROM
UNIVERSIDAD DE BURGOS<br>Universidad de Burgos (UBU)	Spain	Risk assessment of enteric viruses along the food chain and in the population	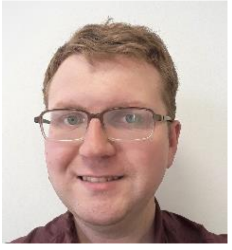<br>Kevin Hunt	Ireland
			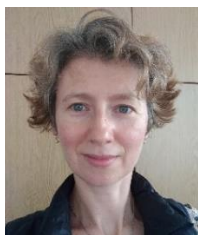<br>Monika Trząskowska	Poland
Universidad Politécnica de Cartagena<br>Universidad Politecnica de Cartagena (UPCT)	Spain	Training in tools to develop Risk ranking and Quantitative microbial risk assessment along the food chain of Spanish products	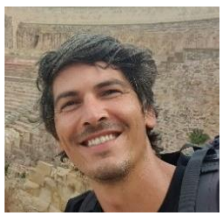<br>Alessandro Zambon	Italy
UNIVERSITÀ DI PARMA<br>Università degli Studi di Parma (UNIPR)	Italy	Changes in terms of risk/benefit of shifting diets towards healthier and more sustainable dietary models	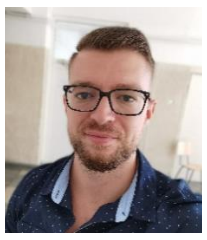<br>Octavian Mihalache	Romania
			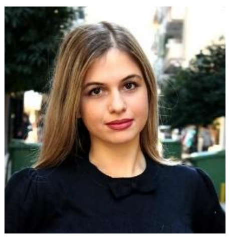<br>Aliki Kalmpourtzidou	Greece
UBO université de bretagne occidentale<br>LUBEM Laboratoire Universitaire de Biodiversité et d'Écologie Microbienne<br>Université de Bretagne Occidentale - (Laboratoire Universitaire de Biodiversité et Ecologie Microbienne) (UBO)	France	Innovative in vitro approaches to toxicological investigations of mycotoxins effects	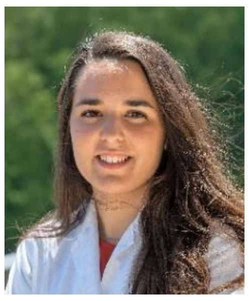<br>Beatriz Arce López	Spain

HOSTING ORGANISATION	COUNTRY	WORK PROGRAMME	FELLOW	FROM
University of Granada (INYTA/UGR)	Spain	Microbiota analysis for risk assessment of xenobiotics and its potential impact on dysbiosis and endocrine	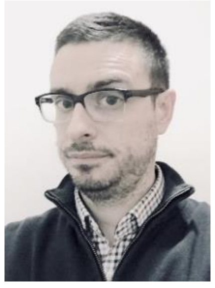 **Antonios Ampatzoglou**	Greece
			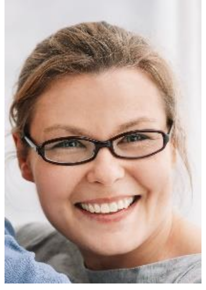 **Agnieszka Gruszecka-Kosowska**	Poland
University of Maribor & Josef Stefan Institute (UM & JSI)	Slovenia	Implementation of matrix effects into chemical food contaminant risk assessment	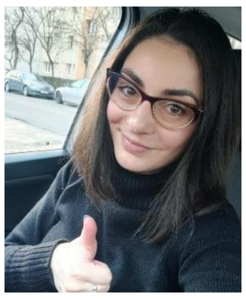 **Ana-Andreea Cioca**	Romania
University of Veterinary Medicine Budapest (UVMB)	Hungary	Emerging risk identification by applying data analytical tools	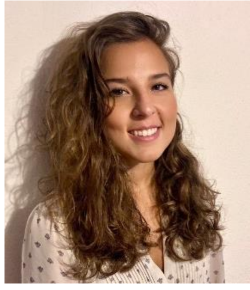 **Elisa Palmas**	Italy
