# Supplementary material for: Food Risk Analysis: Towards a Better Understanding of “Hazard” and “Risk” in EU Food Legislation
Source: Foods. 2023 Jul 27;12(15):2857. doi: 10.3390/foods12152857 (PMC10418315; doi:10.3390/foods12152857)
Supplement: Supplementary file 1 [file foods-12-02857-s001.zip › Supplementary Material 3/Instructions.pdf]

Dear expert,

Thank you for offering your support in this EU-FORA research study. The research aims to identify and analyze inconsistencies in the translation of some food regulations reffering to risk assessment. The focus was on finding wrongful/incorrect translations of the most important terms, such as „hazard", risk", including „risk analysis", „risk assessment", etc). To better understand food risk assessment and the difference between „hazard" and risk" we should have accurate translations from English to UE member state languages.

Here are some instructions on how to fill in the Excel containing inconsistencies in the translation of „hazard", „risk", etc.

1. Please refresh your memory with the definition of „hazard" and „risk" in Regulation (EC) 178/2002 and Regulation (EC) 625/2019 available here:

Regulation (EC) 178/2002: page 7-8

https://eur-lex.europa.eu/legal-content/EN/TXT/PDF/?uri=CELEX:32002R0178&from=SL

Regulation (EC) 625/2019: page 20

https://eur-lex.europa.eu/legal-content/EN/TXT/PDF/?uri=CELEX:32017R0625&from=SL

2. Please check the definitions also in your native language and observe the words used for the translation. You can simply replace EN with the two letter which correspond to your native language.

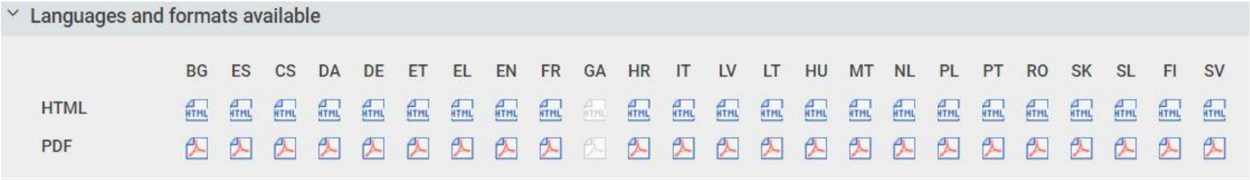

3. In Excel you will find 5 different sheets with different regulation titles (e.g. REG 178-2002, REG 852-2004, etc). Please go through each sheet and fill in column G. In column F you will find a comment/question based on which you should construct reply.

4. Please inform me if you believe that the words used for „hazard" and „risk" in your native language (definitions Regulation (EC) 178/2002 and Regulation (EC) 625/2019) are accurate? Is there a clear difference between „hazard" and „risk"? Please add the answeres in the email body when you reply.

Filling in should probably take around **45 min – 1 h** of your time. If possible, please return the Excel containing your reply to **anaandreeacioca@yahoo.com** by **February 15th 2022.**

Thank you for your time and consideration!

Ana-Andreea Cioca
