# Supplementary material for: Food Risk Analysis: Towards a Better Understanding of “Hazard” and “Risk” in EU Food Legislation
Source: Foods. 2023 Jul 27;12(15):2857. doi: 10.3390/foods12152857 (PMC10418315; doi:10.3390/foods12152857)
Supplement: Supplementary file 1 [file foods-12-02857-s001.zip › Supplementary Material 1.pdf]

Software Tool Description (development, function, examples)

This tool has been developed in Java with the aid of the Spring Boot framework for the processing side. Other frameworks and libraries that have been used are:

- Thymeleaf – for the design of the webpage where we can use the actual program
- Java string similarity library: https://github.com/tdebatty/java-string-similarity - for detecting the similarity between keywords and, more exactly, finding the keywords in the articles we want to compare
- Apache PDFBox : https://pdfbox.apache.org/ - for manipulating the PDF articles

The user has to configure in the main page of the tool the 2 articles he wants to compare and also a series of keywords for each article that the program will search for and highlight in each document

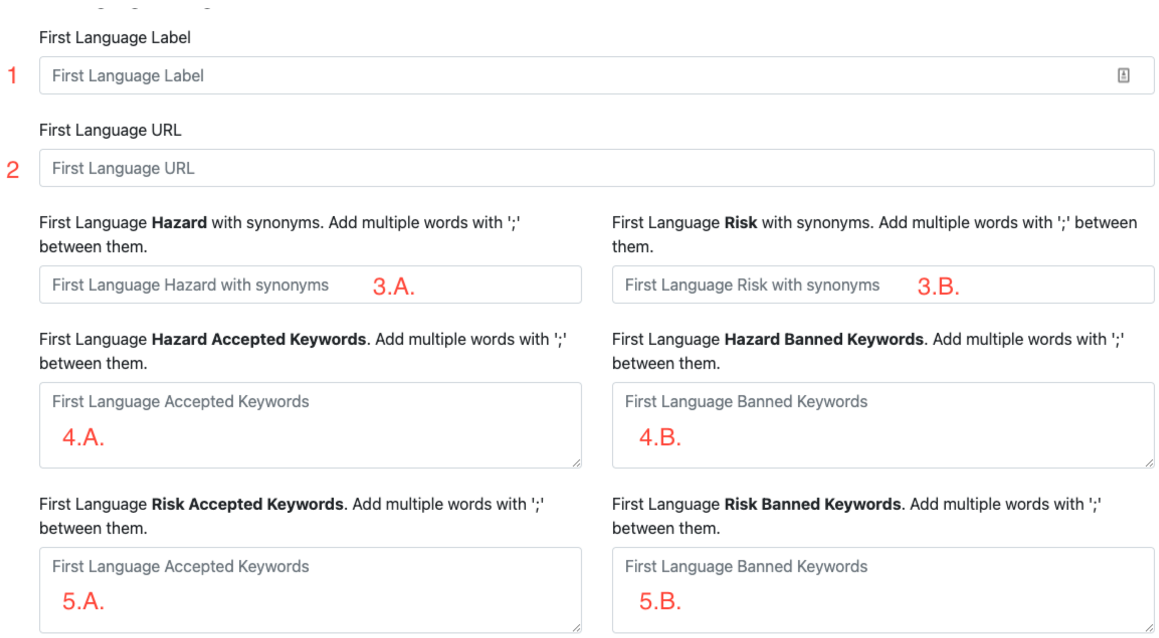

We will take as an example the first language configuration, and the second one will be exactly the same.

1. A label to remember which language is which
2. The URL which is pointing to the PDF that it is containing the article
3.A. All the synonyms of the "Hazard" keyword separated by ";" which the program will attempt to find inside the PDF article
3.B. All the synonyms of the "Risk" keyword separated by ";" which the program will attempt to find inside the PDF article
4.A. All the accepted words separated by ";" for the "Hazard" keyword. Basically, it will attempt to find inside the PDF article all words that match 100% the words defined here; this is useful if we know exactly some words that we want to include and not use for them the similarity algorithm described below since the similarity algorithm doesn't produce always accurate results, especially for more complex languages
4.B. All the banned words separated by ";" for the "Hazard" keyword. Basically, it will attempt to ignore inside the PDF article all words that match 100% the words defined here; this is useful if we know exactly some words that we want to exclude and not use for them the similarity algorithm

described below since the similarity algorithm doesn't produce always accurate results, especially for more complex languages
5.A. All the accepted words separated by ";" for the "Risk" keyword. Basically, it will attempt to find inside the PDF article all words that match 100% the words defined here; this is useful if we know exactly some words that we want to include and not use for them the similarity algorithm described below since the similarity algorithm doesn't produce always accurate results, especially for more complex languages
5.B. All the banned words separated by ";" for the "Risk" keyword. Basically, it will attempt to ignore inside the PDF article all words that match 100% the words defined here; this is useful if we know exactly some words that we want to exclude and not use for them the similarity algorithm described below since the similarity algorithm doesn't produce always accurate results, especially for more complex languages

After the user has completed these 2 sections (First and Second Languages Configurations), the user can press on the "Process Differences" button.

The program will then take the following actions:

1. Download the first PDF from the URL configured in the sections above (at point. 2)
2. For each word inside the PDF it will:
    A. Compare it with the list of keywords defined at points 3.A. and 3.B. by using a **custom similarity algorithm** (see below)
    B. If the word is found in one of the lists, if it is a Hazard synonym, it will be highlighted in red and if it's a Risk synonym it will be highlighted in yellow
    C. If the word is not found either in the list of Hazard or the list of Risk synonyms, it will be compared exactly with the words defined in the Accepted / Banned keywords (4.A., 4.B., 5.A., 5.B.)
    D. Again, if the word is found as a Hazard, it will be highlighted in red, otherwise if it will be a Risk, it will be highlighted in yellow
3. Download the second PDF from the URL configured in the sections above
4. Do the same steps for the second PDF as in #2 above
5. Present the 2 PDF documents side by side with the interesting keywords highlighted so the user can compare easily the 2 documents and find differences between them

Custom similarity algorithm

The algorithm is comprised of 13 similarity algorithms. Each of the 13 similarity algorithms basically computes a degree of similarity between the word in the text, and the word that interests us. After, it will attach some weights to each percentage (depending on how accurate the algorithm is) and multiply everything together. If the result is greater than a step of confidence (which is configurable inside the program), then the word is marked as found and can be highlighted accordingly.

<u>Example:</u>
We take the word "risk" and we want to compare it with "risb".
For each similarity algorithm described below, we will take the 2 words and compare the degree of similarity between them, producing a number between 0 and 1.
We will multiply each result with a weight (decided based on how reliable the algorithm is) and multiply everything together like so:

finalPercentage =
0.5 * percentageJaroWinkler +
0.12 * percentageRatcliffObershelp +
0.12 * percentageCosine +
0.1 * percentageSorensenDice +
0.017 * percentageLevenshtein +
0.017 * percentageNormalizedLevenshtein +
0.017 * percentageDamerau +
0.017 * percentageOptimalStringAlignment +
0.017 * percentageLongestCommonSubsequence +
0.017 * percentageNgram +
0.017 * percentageQGram +
0.017 * percentageJaccard +
0.024 * percentageSift4;

If the final percentage is greater than a degree of confidence that we picked (in this case 0.8), then we say that "risk" and "risks" are the same word.
It was necessary to develop this algorithm because, as it can be seen above, if we compare one for one the words, in case of "risk" and "risks", these 2 do not match, but they are the same since risks is the plural of risk.

The 13 algorithms that have been used (with a short description) are:

1. **Levenshtein Distance**
   The Levenshtein distance between two words is the minimum number of single-character edits (insertions, deletions or substitutions) required to change one word into the other.

2. **Normalized Levenshtein**
   This distance is computed as levenshtein distance divided by the length of the longest string. The resulting value is always in the interval [0.0 1.0] but it is not a metric anymore!

3. **Damerau-Levenshtein**
   Similar to Levenshtein, Damerau-Levenshtein distance with transposition (also sometimes calls unrestricted Damerau-Levenshtein distance) is the minimum number of operations needed to transform one string into the other, where an operation is defined as an insertion, deletion, or substitution of a single character, or a transposition of two adjacent characters.

4. **Optimal String Alignment**
   The Optimal String Alignment variant of Damerau–Levenshtein (sometimes called the restricted edit distance) computes the number of edit operations needed to make the strings equal under the condition that no substring is edited more than once, whereas the true Damerau–Levenshtein presents no such restriction. The difference from the algorithm for Levenshtein distance is the addition of one recurrence for the transposition operations.

5. **Jaro-Winkler**
   Jaro-Winkler is a string edit distance that was developed in the area of record linkage (duplicate detection) (Winkler, 1990). The Jaro–Winkler distance metric is designed and best suited for short strings such as person names, and to detect transposition typos.
   Jaro-Winkler computes the similarity between 2 strings, and the returned value lies in the interval [0.0, 1.0]. It is (roughly) a variation of Damerau-Levenshtein, where the transposition of 2 close characters is considered less important than the transposition of 2

characters that are far from each other. Jaro-Winkler penalizes additions or substitutions that cannot be expressed as transpositions.

6. **Longest Common Subsequence (LCS)**
   The LCS problem consists in finding the longest subsequence common to two (or more) sequences. It differs from problems of finding common substrings: unlike substrings, subsequences are not required to occupy consecutive positions within the original sequences.
   The LCS distance between strings X (of length n) and Y (of length m) is n + m - 2 |LCS(X, Y)| min = 0 max = n + m
   LCS distance is equivalent to Levenshtein distance when only insertion and deletion is allowed (no substitution), or when the cost of the substitution is the double of the cost of an insertion or deletion.

7. **N-Gram**
   Normalized N-Gram distance as defined by Kondrak, "N-Gram Similarity and Distance", String Processing and Information Retrieval, Lecture Notes in Computer Science Volume 3772, 2005, pp 115-126.
   The algorithm uses affixing with special character '\n' to increase the weight of first characters. The normalization is achieved by dividing the total similarity score the original length of the longest word.

8. **Q-Gram**
   Q-gram distance, as defined by Ukkonen in "Approximate string-matching with q-grams and maximal matches" http://www.sciencedirect.com/science/article/pii/0304397592901434
   The distance between two strings is defined as the L1 norm of the difference of their profiles (the number of occurences of each n-gram): SUM(|V1_i - V2_i|). Q-gram distance is a lower bound on Levenshtein distance, but can be computed in O(m + n), where Levenshtein requires O(m.n)

9. **Cosine similarity**
   The similarity between the two strings is the cosine of the angle between these two vectors representation, and is computed as V1 . V2 / (|V1| * |V2|)

10. **Jaccard index**
    Like Q-Gram distance, the input strings are first converted into sets of n-grams (sequences of n characters, also called k-shingles), but this time the cardinality of each n-gram is not taken into account. Each input string is simply a set of n-grams. The Jaccard index is then computed as |V1 inter V2| / |V1 union V2|.

11. **Sorensen-Dice coefficient**
    Similar to Jaccard index, but this time the similarity is computed as 2 * |V1 inter V2| / (|V1| + |V2|).

12. **Ratcliff-Obershelp**
    Ratcliff/Obershelp Pattern Recognition, also known as Gestalt Pattern Matching, is a string-matching algorithm for determining the similarity of two strings. It was developed in 1983 by John W. Ratcliff and John A. Obershelp and published in the Dr. Dobb's Journal in July 1988

13. **SIFT4**

SIFT4 is a general-purpose string distance algorithm inspired by JaroWinkler and Longest Common Subsequence. It was developed to produce a distance measure that matches as close as possible to the human perception of string distance. Hence it takes into account elements like character substitution, character distance, longest common subsequence etc. It was developed using experimental testing, and without theoretical background.
