# Supplementary figures and images for: Food Risk Analysis: Towards a Better Understanding of “Hazard” and “Risk” in EU Food Legislation
Source: Foods. 2023 Jul 27;12(15):2857. doi: 10.3390/foods12152857 (PMC10418315; doi:10.3390/foods12152857)

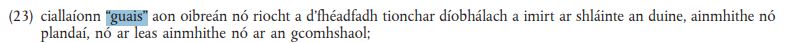

Supplement: Supplementary file 1 [file foods-12-02857-s001.zip › Supplementary Material 3/GA/hazard - GA.JPG]

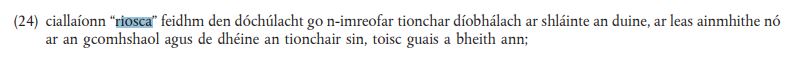

Supplement: Supplementary file 1 [file foods-12-02857-s001.zip › Supplementary Material 3/GA/risk - GA.JPG]

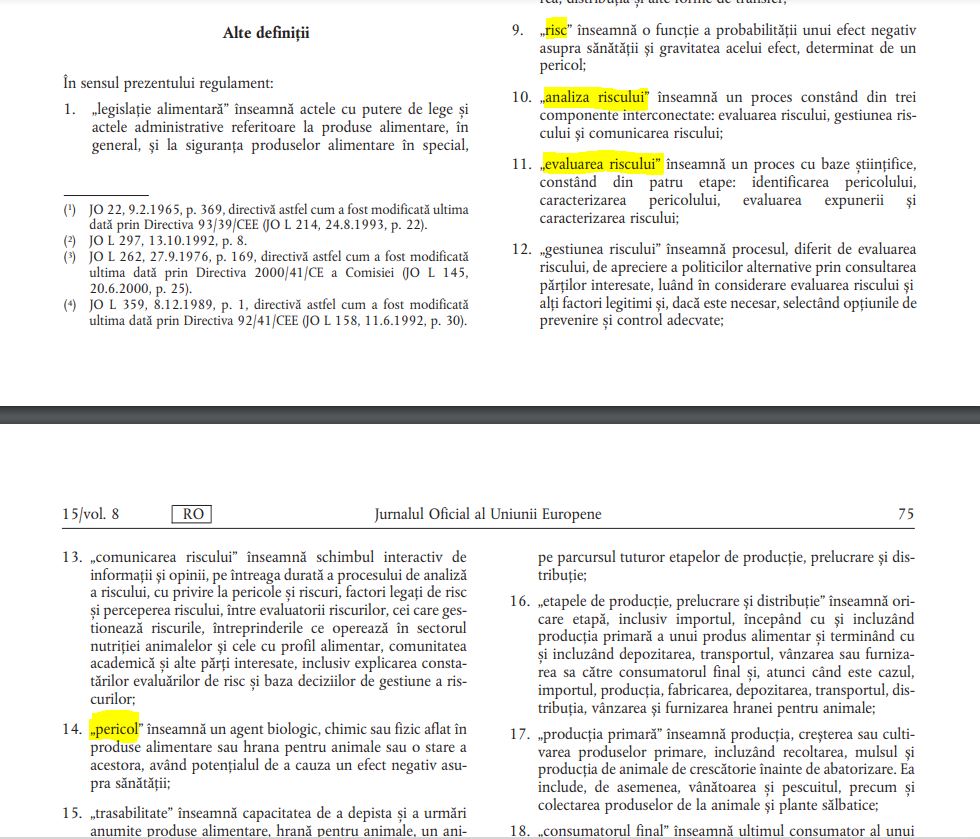

Supplement: Supplementary file 1 [file foods-12-02857-s001.zip › Supplementary Material 3/RO/Reg 178 - RO definitions hazard and risk.JPG]

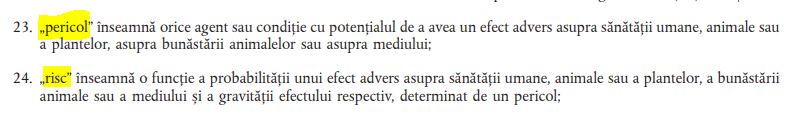

Supplement: Supplementary file 1 [file foods-12-02857-s001.zip › Supplementary Material 3/RO/Reg 625 - RO definitions hazard and risk.JPG]
